# Recruiting Persons With Dementia: A Systematic Review of Facilitators, Barriers, and Strategies

**DOI:** 10.1177/15333175241276443

**Published:** 2024-08-13

**Authors:** Julian Hirt, Thomas Beer, Stefano Cavalli, Stefano Cereghetti, Elia R. G. Pusterla, Adelheid Zeller

**Affiliations:** 1 Department of Health, 112888 Eastern Switzerland University of Applied Sciences, St.Gallen, Switzerland; 2 Pragmatic Evidence Lab, Research Center for Clinical Neuroimmunology and Neuroscience Basel (RC2NB), 638593 University Hospital Basel and University of Basel, Basel, Switzerland; 3 Centre of Competence on Ageing, Department of Business Economics, Health and Social Care, 638593 University of Applied Sciences and Arts of Southern Switzerland (SUPSI), Manno, Switzerland

**Keywords:** dementia [MeSH], nursing research [MeSH], health services research [MeSH], patient selection [MeSH], recruitment

## Abstract

Study recruitment of persons with dementia is challenging. We aimed to assess facilitators, barriers, and strategies to identify and approach persons with dementia for recruitment to dementia care studies. We systematically searched MEDLINE/PubMed, CINAHL, Web of Science, and other sources (ORRCA [Online Resource for Research in Clinical triAls]; pertinent evidence syntheses; citation searching) and narratively summarised the results (PROSPERO CRD42022342600). Facilitators and barriers consisted of “characteristics of participants, researchers, clinical contact persons”, “study characteristics”, and “communication with participants”. The highest number of participants were recruited by study information in electronic and print formats, as well as by networking and collaboration. Advertisements proved to be the most expensive way of recruitment. There is limited evidence on the impact of recruitment strategies to identify persons with dementia for recruitment to dementia care studies. Our analysis of facilitators and barriers may inform research teams in designing strategies to identify persons with dementia for recruitment purposes.

## Significance Statement

A systematic search of the literature yielded 18 studies assessing facilitators, barriers and strategies to identify persons with dementia for recruitment to dementia care studies. Our review revealed important barriers to identifying and approaching persons with dementia for recruitment (e.g., “characteristics of persons involved”; “study-related communication measures”). We found limited evidence on the efficacy of the identified recruitment strategies. Our results provided a basis for developing strategies and overcoming barriers to identifying and approaching persons with dementia for recruitment to dementia care studies.

## Introduction

Worldwide, dementia is a major cause of disability and care dependency among older adults. It has an impact on memory, cognition, function, and behaviour. Globally, approximately 47 million people are affected. This corresponds to about 5% of the world’s population aged 65 and older.^1^ Thus, the World Health Organization recognized dementia as a public health priority.^2^ Pharmacological interventions may have a preventive or slowing effect on the progress of the disease.^3^ However, as dementia is incurable, the primary objective is to support the ability to perform activities of daily living, to maintain quality of life, and to ensure access to health and long-term care services.^4^

Dementia care researchers seek to develop and evaluate targeted health interventions to improve quality of life in persons with dementia.^5^ To gain a deeper understanding of the individual physical, cognitive, psychological, social, and emotional needs of persons with dementia and to assess the benefit of dementia-specific interventions, it is essential that persons with dementia themselves take part in research.^6,7^

Recruiting persons with dementia is challenging in many ways. They often have limited opportunities to report their experiences and to share their personal insights.^8^ Furthermore, they may have a reserved attitude toward research participation, for example due to unfamiliarity with the research community and a lack of confidence in the potential of meaningful or beneficial research results.^9^ Motivating factors to participate in research include hope for cure or symptom improvement, incentives, or a personal desire to contribute to (medical) research.^9,10^ For researchers, it may be difficult to identify and approach persons with dementia through public channels. Recruitment is regarded as particularly demanding when persons with dementia are cared for by others, are seldom active outside their home or become increasingly self-isolated.^8,11-13^ Furthermore, clinical staff may have a negative attitude towards involving persons with dementia in research and therefore may advice against participation.^9^ Due to recruitment challenges, there is a risk that persons with dementia are excluded from research. As an alternative, people with similar impairments or relatives are recruited to gain insight into the perspectives of persons with dementia.^14,15^

Research on recruitment strategies in dementia care studies has predominantly focussed on specific trial purposes (e.g., clinical drug trials^16,17^ or prognostic studies),^18^ particular populations (e.g., ethnic groups^19,20^ or individuals from underrepresented backgrounds)^21^ or specific contexts (e.g., countries^22^ or health care sectors).^23^ In contrast, Davis and Bekker in a recent review explored best practices for recruiting persons with dementia in general, including involvement in dementia care studies.^9^ They concluded that the most effective recruitment strategies consist of multiple recruitment methods, collaboration with community partners, and incentives. However, the review covered a broad spectrum of dementia studies. It considered not only persons with dementia but also with cognitive impairment. Thus, a comprehensive review of strategies to identify persons with dementia for recruitment to dementia care studies of any empirical study design is lacking. Such a review may inform researchers to find effective ways to involve persons with dementia in upcoming studies.

We therefore aimed (i) to investigate facilitators and barriers to identifying and approaching persons with dementia for recruitment to dementia care studies and (ii) to determine the effectiveness of recruitment strategies.

Our review questions were:

What are the facilitators and barriers to identifying and approaching persons with dementia for recruitment to dementia care studies? (review question 1).

What are effective strategies to identify persons with dementia for recruitment to dementia care studies? (review question 2).

## Methods and Analysis

We registered our systematic review in the Prospective Register of Systematic Reviews (PROSPERO CRD42022342600). To structure this report, we used (where applicable) the updated Preferred Reporting Items for Systematic review and Meta-Analysis (PRISMA 2020).^24^

### Eligibility Criteria

We included empirical qualitative, quantitative, and mixed-methods studies explicitly aiming at (i) the investigation of facilitators and barriers to identifying and approaching persons with dementia for recruitment to dementia care studies and/or (ii) the evaluation of recruitment strategies to identify persons with dementia for recruitment to dementia care studies. We considered journal articles without restriction on publication year, language, setting, intervention (specific to review question 2) or outcome (specific to review question 2). To address our first review question on facilitators and barriers from multiple perspectives, we did not restrict the study population. We took into consideration studies involving persons with dementia who reported on their (potential) recruitment experience. Furthermore, we included studies in which researchers, care professionals or family members described their experience of recruiting persons with dementia to participate in dementia care studies. Regarding our second review question on strategies, we included studies only under the condition that 75% or more of the study sample had dementia, if the study topic was not dementia (e.g., stroke, Parkinson’s disease, or aging). We considered opinion-based journal articles (commentaries, editorials, lessons learned) if the authors reflected on recruitment issues based on their research experience. Conference proceedings, abstract-only publications, and studies on registries intending to collect data on people without dementia (i.e., healthy volunteers) were excluded.

We defined “dementia care studies” as care-related studies (i.e., health services research, nursing research, allied health research) focussing on the physical, psychological, emotional, cognitive, and social support of persons with dementia. Research related to pharmacological treatment, genetics, prevention, diagnostics or screening remained unconsidered.

### Information Sources and Search Strategy

We searched MEDLINE/PubMed, CINAHL, and the Web of Science Core Collection on June 27, 2022 (Supplemental Appendix 1).^25^ As supplementary sources, we searched ORRCA (Online Resource for Research in Clinical triAls) on August 8, 2022 (“dementia” in titles) and relevant evidence syntheses for additional studies (identified during database and supplementary searches). In addition, we conducted multiple iterations of forward (citing articles) and backward (cited articles) citation searches of all eligible studies using Scopus until no further relevant studies were identified.^26^

Our search strategy included both topics (“recruitment” and “dementia”). It was informed by (i) available methodological reviews on the recruitment of multiple populations^27,28^ and (ii) by dementia-related terminology (free text terms and controlled vocabulary, when applicable). Our experience of conducting topical reviews^6,25,29-32^ informed and validated our search strategy.

### Study Selection and Data Extraction

Titles and abstracts were screened by an experienced reviewer (JH) to determine the potential eligibility of records. In case of uncertainty, he consulted a second reviewer (AZ) for confirmation. Full texts were screened by two experienced reviewers independently and in duplicate (JH and AZ). We utilized Citavi for reference and full-text management. To select studies, we used Rayyan.^33,34^

For data extraction, we developed and piloted a standardized spreadsheet. We extracted bibliographic information of all eligible studies (i.e., authors, publication year) as well as country, study aim, study design, setting (e.g., hospital, nursing home), sample size, and results. Concerning our first review question on facilitators and barriers, we extracted information on the population involved (i.e., persons with dementia, researchers, care professionals, and family members), data collection, and methods of analysis. Regarding our second review question on strategies, we extracted information on type of strategy and comparison, outcome and measurement, and results.

### Critical Appraisal

To critically appraise the quality of the included studies, we used the Mixed Methods Appraisal Tool (MMAT).^35^ MMAT can be applied to multiple study designs such as interventional, observational, qualitative, and mixed-methods studies. The assessment contains overall quality criteria (e.g., if a study contains a clear research question/aim) as well as basic quality criteria related to each of the study designs mentioned above (e.g., appropriateness of measurement(s) in observational studies or adequate derivation of findings from the data). One reviewer (JH) experienced with the MMAT tool and risk of bias assessment performed the critical appraisal. We critically appraised the study that was relevant to answer our review questions; for example, when survey data was used to assess the recruitment within a randomized trial, we assessed the methodology of the survey (cross-sectional part) and not the randomized trial.

Critical appraisal results were double-checked and confirmed by a second reviewer (AZ). Studies were not excluded based on the critical appraisal results.

### Data Analysis and Synthesis

To answer our first review question, we summarized the results of individual studies using MAXQDA.^36^ One reviewer (JH) identified and coded relevant study results line by line. He categorized codes and tabulated them. A second reviewer (AZ) verified them. We did not use a framework for categorization. For each category, JH formulated a descriptive summary with examples. AZ verified the summary. The aim was to provide information on the category supported by the individual study results.^37^

We tabulated the identified strategies and outcomes to answer our second review question. In a harvest plot, we graphically illustrated recruitment strategies, outcomes (i.e., those that were at least reported twice across eligible studies) and their corresponding results. Afterwards we formulated a narrative summary.^38,39^ JH transferred results not reported in percentages (i.e., recruitment rates) or numbers (i.e., recruited participants or cost) into a low/moderate/high continuum and AZ verified it. We narratively summarized information on study and sample characteristics, using numbers and percentages.

## Results

### Literature Search and Selection Process

**Figure 1.**
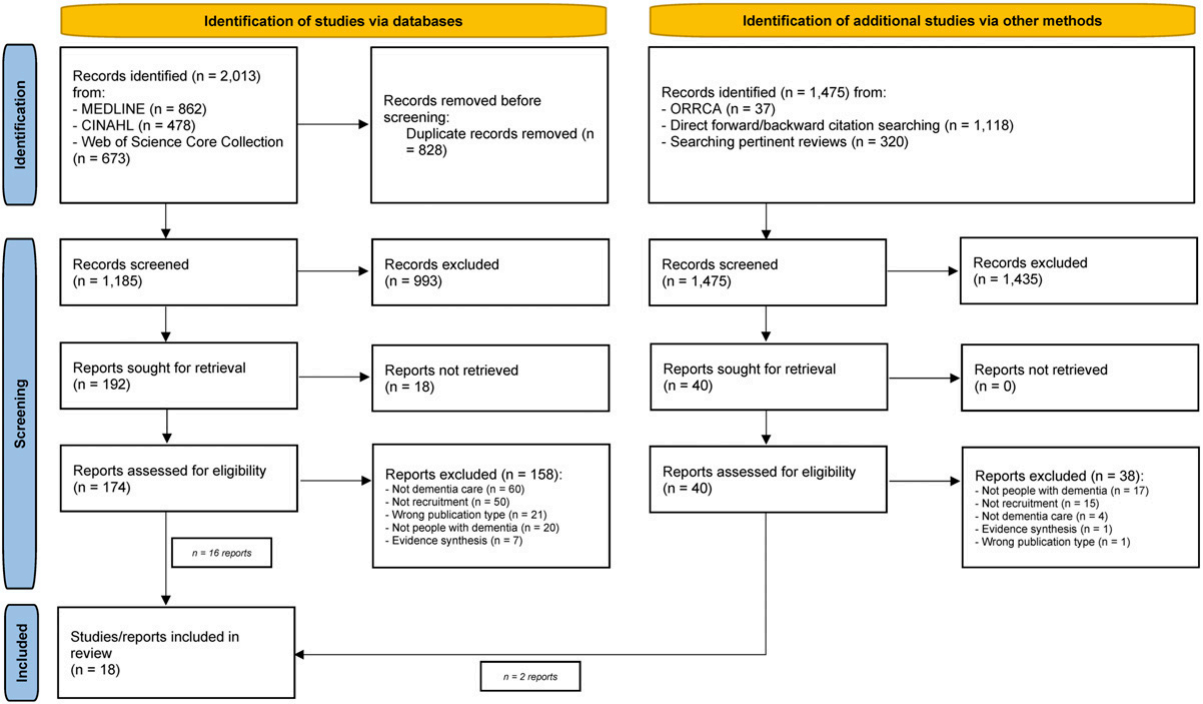
Literature search and study selection process (PRISMA 2020 flow diagram). Abbreviations: CINAHL, Cumulative Index to Nursing and Allied Health Literature; MEDLINE, Medical Literature Analysis and Retrieval System Online; ORRCA, Online Resource for Research in Clinical triAls.

Our database and supplementary searches yielded 3528 records. We identified 18 eligible studies. Eleven of these were relevant to our first review question,^40-50^ ten were appropriate to answer our second review question^42,47,48,51-57^ (Figure 1; Supplemental Appendix 1).

### Facilitators and Barriers

Almost half of the eleven studies that concerned our first review question (Table 1; Supplemental Appendix 2) were conducted in the United States (n = 5; 45%), had a qualitative study design (n = 5; 45%), and took place in the community or home setting (n = 5; 45%). The severity of dementia was predominantly described as “capacity to participate” (n = 3; 27%) or was not reported (n = 4; 36%). Facilitators and barriers were most often reported by formal and/or informal caregivers n = 8; 72%), by persons with dementia (n = 6; 55%), or by researchers (n = 3; 27%). Data collection was mostly based on interviews (n = 7; 64%) or surveys (n = 3; 27%). To analyse data, content analysis (n = 6; 55%) or descriptive statistics (n = 3; 27%) were applied. The sample size of the studies ranged between 7 and 206 (median: 25).

**Table 1.** Characteristics of Included Studies.^a^

	Review Question #1 (n = 11)	Review Question #2 (n = 10)
	n (%)	n (%)
Year (range, median)	2001-2022, 2020	2014-2022, 2019
Country		
US	5 (45)	4 (40)
UK	2 (18)	3 (30)
Canada	2 (18)	—
The Netherlands	1 (9)	2 (20)
Australia	1 (9)	1 (10)
Study design		
Cross-sectional study	4 (36)	8 (80)
Qualitative study	5 (45)	1 (10)
Mixed-methods study	2 (18)	—
Various^b^	—	1 (10)
Setting^c^		
Community/home	5 (45)	7 (70)
Nursing or care home	4 (36)	1 (10)
Hospital	2 (18)	—
Memory clinic	1 (9)	—
Holiday accommodation	—	1 (10)
Various^b^	—	1 (10)
Not specified	1 (9)	—
Sample: severity of dementia		
Based on assessment score	2 (18)	2 (20)
Not specified	1 (9)	3 (30)
Capacity to participate	3 (27)	—
Mild to moderate	—	3 (30)
Capacity to consent	1 (9)	—
Various^b^	—	1 (10)
NA	4 (36)	1 (10)
Sample: type/perspective^c^		
Caregivers (formal and/or informal)	8 (72)	NA
People with dementia	6 (55)	NA
Researchers	3 (27)	NA
Clinicians	1 (9)	NA
Representatives	1 (9)	NA
Sample: n individuals (range, median)	7-206, 25	25-1,275, NA^d^
Data collection methods^c^		
Interview	7 (64)	NA
Survey	3 (27)	NA
Document analysis	2 (18)	NA
Field notes	1 (9)	NA
Literature review	1 (9)	NA
Data analysis methods^c^		
Content or thematic analysis	6 (55)	NA
Descriptive statistics	3 (27)	NA
No information	4 (36)	NA

Notes: NA, Not applicable or data has not been extracted; —, No cases counted.

Abbreviations: UK, United Kingdom; US, United States.

^a^Individual study characteristics are provided in Supplemental Appendix 2;

^b^Multiple studies were evaluated^51^;

^c^More than one category possible;

^d^Various number of participants within a study.^51^

We assessed the sampling strategy in cross-sectional studies and justification of the design in all included qualitative studies that addressed the first review question as adequate. Half of the cross-sectional studies did not provide details on data collection instruments and thus, we could not assess the appropriateness of the measurements used throughout all studies. We assessed the presentation of data in a descriptive way (narrative and/or in numbers) as appropriate throughout all eleven studies that addressed the first review question (Supplemental Appendix 3).

Eight categories on facilitators and barriers to identifying and approaching persons with dementia for recruitment to dementia care studies emerged which we describe in the following sections (see Table 2 and Supplemental Appendix 2 for details).

**Table 2.** Identified Barriers and Facilitators to Identifying and Approaching People With Dementia for Recruitment to Dementia Care Studies.

Categories	Facilitators	#	Barriers	#
Persons involved				
Participants involved	Personal learning desires	^46^	Denial of diagnosis	^45^
	Happiness to help	^46^	Clinic appointment being already overwhelming	^45^
	Do something for society	^47^	Periods of uncertainty	^43^
	Financial incentives	^46^	Expected participation burden	^47^
			Lack of time of the caregiver	^47^
Researchers involved	Designated team with clinically involved persons	^42^	—	
	Positive personal attributes	^43^		
	Communication skills	^43^		
	Team discussions of attitudes and beliefs prior to approaching families for consent	^50^		
Clinical contact persons involved	Collaboration with clinicians that are linked to the setting	^42^	Management staff turnover	^42^
	Research training for care home staff	^42^	Research refusal by care home organization	^42^
	Willingness of care home managers	^44^	Memory clinic staff’s lack of understanding of research recruitment	^45^
	Dedicated recruitment time for care home staff	^44^	Study workload and resource need (space, trained staff, and time)	^45^
			Perceived conflict of interest between research recruitment and provision of care	^45^
			Need for rest and limited benefit expressed by family caregivers	^50^
Study characteristics				
	Possibility to exercise during the intervention	^47^	Studies that require a large time commitment from care home staff	^42^
	Home-based intervention	^47^	Mismatch of research protocol and care home’s mode of operation	^42^
	Offer multiple data collection methods, i.e., interviews over time and in different formats (e.g., individual, group)	^49^	Long distances to research centers	^45^
			Intensity of the intervention being studies	^47^
			Transportation needs and related cost	^45^
Communication with participants				
Content of communiation with participants	Participation in and withdrawal from the study has no impact on care received	^40,50^	Lack of study information, expectations of participants (i.e., time commitment), and potential benefits of participation	^45^
	Description of the study including study information, potential benefits, study strengths, and limitations	^40,42,43^		
	Societal and personal benefit of research participation	^48^		
	Positive experiences of past research participants	^48^		
	Educational communication material that acknowledges the difficulty of certain questions for potential participants	^40^		
	Information about participant preferences (i.e., communication styles, support/access needs)	^49^		
Type of communcation with participants	Clear visibility of research staff (e.g., remove badges that are similar in branding or style to the care staff)	^40^	Lack of understandable study information	^45^
	Principal investigator providing information about the study	^40^		
	Offer the opportunity to follow-up and consent to the study after discharge from the acute illness care setting	^40^		
	Clarify and acknowledge potential participant’s understanding and knowledge of research and personal experience with or concerns about dementia	^40,48^		
	Involve people with dementia (i.e., even if they themselves cannot consent to the study due to lack of decisional capacity)	^40,43^		
	Building a relationship	^43^		
	Study information brochure	^42^		
	Detailed and clear communication for study description	^43^		
	Language that distinct between care and research	^40^		
	Avoid medical and scientific jargon	^48^		
	Explanatory chains to build understanding of the research process	^48^		
	Making sense of data through social maths	^48^		
	Choose visuals and stories that represent equity in all aspects of the research	^48^		
Caregiver involvement in communcation with participants	Address all caregivers present in the room (if more than one)	^40^	—	
	Involving relatives in the research process	^42^		
	Offer time to potential participants to consult with important decision-makers about participating in the study	^40^		
	Preparing information for caregivers on how they could be supportive during data collection (e.g., during interviews)	^49^		
	Work with nursing staff to determine the most appropriate time for the study team to approach a potential participant (i.e., to anticipate and minimize disruptions during conversation)	^40^		
	Confirm that the potential participant has decisional capacity or (proxy) decision maker	^40^		
Setting of communication with participants	Meet potential participants where they are (e.g., community service centres, physician’s office)	^41^	Limited time within clinic assessment appointment to introduce studies	^45^
	In-person meet	^46^		
	Offer to speak to family members at the nursing home or at home	^50^		
	Recruitment calls to caregivers at non-typical working hours (i.e., in the evening or on Sunday)	^50^		

Abbreviations: #, Reference number; —, No barriers have been identified.

#### Participants Involved

This category includes facilitators and barriers related to the personal and social situation of potential participants. The burden of dementia is also considered in this category. With regard to facilitating conditions, the following picture emerged: Persons with dementia are more likely to participate in dementia care studies if they have a personal desire to learn, a sense of purpose in helping other people with dementia, and a desire to contribute to society. Financial incentives also play a role. We identified the following barriers: Persons with dementia denied their diagnosis, felt overwhelmed by clinic appointments, experienced periods of uncertainty, perceived an expected burden associated with participation, and faced time constraints due to caring responsibilities.

#### Researchers Involved

This category comprises facilitators related to personal, social and team characteristics. A designated team of clinically experienced individuals with positive personal attributes and strong communication skills is required. The team should be engaged in discussions about attitudes and beliefs before approaching families for consent. This implies a thoughtful and sensitive approach that can evoke trust in potential participants and their families. No barriers were identified in this category.

#### Clinical Contact Persons Involved

This category contains facilitators and barriers related to care settings, such as memory clinics or home care services. Effective collaboration with clinicians associated with the setting proved to be a facilitator. Research training for nursing home staff, willingness of nursing home managers to support recruitment efforts, and allocation of dedicated recruitment time for nursing home staff were favourable for recruitment. Barriers include high turnover of management staff, home care organisations refusing research participation, memory clinic staff without understanding of research recruitment, workload and resource requirements associated with conducting research (space, trained staff, time), perceived conflicts of interest between research recruitment and care provision, family carers’ need for rest, and limited perceived benefit.

#### Study Characteristics

This category includes facilitators and barriers related to specific features and aspects of the study. Providing opportunities for participants to exercise during the intervention have a positive effect. So did implementing home-based interventions. We found that multiple data collection methods, such as interviews conducted over time and in different formats (e.g., individual or group settings) are beneficial. Significant time investment on part of staff, a mismatch between the research protocol and the operational practices of care homes, long distances to research centres, the intensity of the intervention, transportation needs and costs for participants proved to be obstacles.

#### Content of Communication with Participants

This category contains facilitators and barriers related to communicating with potential participants. The following aspects proved to be supportive: ensuring that participation in the study and withdrawal from the study do not affect care, providing a comprehensive description of the study (including information, potential benefits, strengths and limitations), highlighting the societal and personal benefits of research participation, drawing on positive experiences of previous research participants, using educational communication materials that acknowledge the difficulty of certain questions, and taking into account participants’ preferences regarding communication styles and support/access needs. Barriers include lack of adequate study information, participant’s expectations (e.g., time commitment) and clear communication of the potential benefits of participation.

#### Type of Communication with Participants

This category includes facilitators and barriers related to specific communication methods for contacting potential participants. The following aspects proved to be beneficial: Clear visibility of research staff, principal investigators providing information about the study, offering follow-up and consent after discharge from acute care settings, acknowledging knowledge about dementia, showing interest in personal experiences or concerns related to dementia, involving persons with dementia even with diminished decisional capacity, building a relationship with participants, providing detailed and clearly structured study information brochures, offering detailed and clear consent forms, building rapport with participants, providing study information leaflets, offering a detailed and clear study description, using a language distinguishing between care and research, avoiding medical and scientific jargon, providing chains of explanation to make the research process understandable, using social mathematics to make sense of data, and choosing visuals or stories representing equity in all aspects of the research. Lack of understandable trial information was the only barrier we identified.

#### Caregiver Involvement in Communication with Participants

This category comprises facilitators related to the role of caregivers in facilitating communication between potential research participants and the research team. We found the following facilitators: addressing all caregivers present in the room, actively involving relatives in the research process, providing time for potential participants to consult with key decision-makers about participation, preparing information for caregivers on how they can be supportive during data collection (e.g., during interviews), working with caregivers to determine the most appropriate time for the study team to approach a potential participant (with the aim to minimise irritation), confirming that the potential participant has decision-making capacity or identifying a proxy decision-maker, if needed. We identified no barriers in this category.

#### Setting of Communication with Participants

This category includes facilitators and barriers related to locations or contexts of communication with potential research participants. The following aspects proved to be favourable: meeting potential participants where they are (e.g., in community service centres), conducting face-to-face meetings, offering to speak to family members (either in the nursing home or in their own homes), and recruitment calls for family caregivers at atypical working times (e.g., evenings, on Sunday). The only barrier we identified was limited time during appointments to introduce the issue of participation in dementia care studies.

### Strategies

The majority of the ten included studies answering our second review question (Table 1; Supplemental Appendix 2) were conducted in the United States (n = 4; 40%) or in the United Kingdom (n = 3; 30%). They had an observational cross-sectional study design (n = 8; 80%) and took place in the community or home setting (n = 7; 70%). The severity of dementia was predominantly described as “mild to moderate” (n = 3; 30%) or not specified (n = 3; 30%). The sample size of the included studies ranged between 25 and 1275 (median sample sizes are not available due to varying participant numbers within studies).^51^

**Figure 2.**
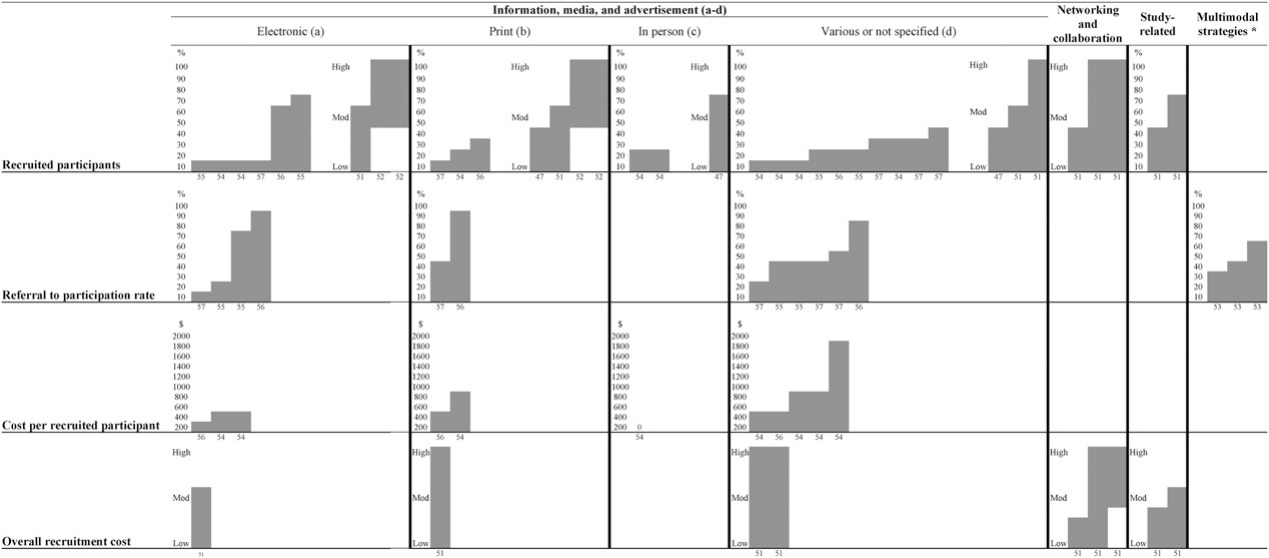
Harvest plot showing results of commonly used outcomes of strategies to identify people with dementia for recruitment to dementia care studies. Note 1: For the outcome *recruited participants*, the bar height represents the percentage number of recruited participants when numbers were given (1-100% categorized in steps of 10, e.g., 57% = 50-60%) or a low/moderate/high-continuum based on authors’ expressions; for the outcome *referral to participation rate*, the bar height represents the percentage referral to participation rate (1-100% categorized in steps of 10, e.g., 57% = 50-60%); for the outcome *cost per recruited participant*, the bar height represents absolute cost per participant illustrated on a scale that reflects the resulted range ($0-1719 in steps of 200, e.g., $480 = 600$); for the outcome *overall recruitment cost*, the bar height represents the absolute overall recruitment cost on a low/moderate/high-continuum based on authors’ expressions. Note 2: Several study outcomes and results are not shown because the outcome has been singularly reported across eligible studies^42,48,53,54^ (for details, see Supplemental Appendix 4). Note 3: The number below the bar represents the study reference. Each investigated strategy/outcome from an individual study is shown in a separate bar. ^*^Multimodal strategies cover several individual recruitment strategies of which results have not been reported separately. Abbreviations: Mod, Moderate.

All included cross-sectional studies had a sampling strategy that we assessed as relevant to address the research question, i.e., the source of sample was relevant to or directly taken from persons with dementia, and we assessed the measurements used and presentation of data in all the included cross-sectional studies as appropriate. However, we assessed the fact that no information on non-responders was provided as a major weakness of the included cross-sectional studies. Thus, if the sample was representative of the target population remained unclear (Supplemental Appendix 3).We clustered the identified strategies into three categories: (i) study-related, (ii) related to study information, media, and advertisement, and (iii) related to networking and collaboration. Furthermore, we identified multimodal recruitment strategies. They covered several individual recruitment strategies without reporting the results of individual strategies However, we did not report the results of individual strategies separately (Figure 2). Details on identified strategies, outcomes, and related results are included in Supplemental Appendices 2 and 4.

All strategies, outcomes, and corresponding results are illustrated in a harvest plot (Figure 2) – except for four studies^42,48,53,54^ reporting on outcomes that were singularly presented across studies. Electronic information and media (direct mailing of potential participants) (57%)^56^ and using the National Health Service Trust database and/or the Join Dementia Research database (62%)^55^ reached the highest number of recruited participants. This was confirmed by studies reporting a considerable recruitment increase (i.e., after a Facebook post and a newsletter by a patient representatives initiative).^52^ These studies did not report their results in percentages. Furthermore, print-based strategies (i.e., news article or advertisement in a local newspaper),^52^ various or not specified interventions (i.e., mass media)^51^ as well as networking and collaboration (i.e., service providers)^51^ also reached higher recruitment numbers.

We identified the following most effective recruitment strategies that achieved the highest participation rate (i.e., ratio of number of referred persons per source/strategy and participating persons):

(1) electronic-based strategies (e.g., Memory Support and Advisory Service database, 67%;^55^ direct mailing, 85%),^56^
(2) print-based strategies (e.g., advertisement in local newspaper, 85%),^56^ and
(3) not specified interventions (e.g., community outreach, 75%).^56^

We found that the lowest cost per recruited participant was reached by clinical and word-of-mouth referral (i.e., zero cost),^54^ and direct mailing ($63).^56^ Advertising generated the highest costs ($1719).^54^ Overall recruitment costs were highest for print-based material (i.e., low to high cost for flyers and posters),^51^ various or not specified interventions (i.e., low to high cost mass media or direct contact with potential participants),^51^ and networking as well as collaboration (i.e., moderate to high cost for partnership with service provider).^51^

## Discussion

Based on 18 studies, our systematic review addresses facilitators, barriers, and strategies for identifying and approaching persons with dementia for recruitment to dementia care studies. As facilitators and barriers we identified characteristics of participants, researchers, clinical contact persons, study characteristics, and the way of contacting potential participants. To recruit persons with dementia, the included studies assessed the impact of information-based strategies, networking and collaboration, study-related aspects, and multimodal strategies. Study information provided by electronic and print-based material reached the highest number of recruited participants. Advertisements proved to be the most expensive strategy.

We identified manifold facilitators and barriers to identifying and approaching persons with dementia for recruitment. These facilitators and barriers highlight the situation of participants, researchers, and clinical contact persons. Previous research revealed that participants’ awareness and restricted access of researchers to the field may influence recruitment.^8^ Our findings confirm that potential benefits of study participation may be unknown to participants.^45^ Without personal motivators and due to missing familiarity with research, recruitment may be difficult.^43^ Aspects that may facilitate recruitment are participants’ positive attitudes and motivation to take part in research. These aspects may be grounded in personal learning desires,^46^ motivation to help,^46^ the intention of contributing to society,^47^ and financial incentives.^9,46^

Persons with dementia may be excluded as study participants when proxy assessments are performed with family or professional caregivers.^6,14,58,59^ Researcher should consider the role of family caregivers when they are interested in recruiting persons with dementia in the domestic setting. We highlighted specific recruitment barriers, for example lack of caregivers’ time,^47^ expected burden from research participation,^47^ concerns about the capacity for participating in research and doubts concerning the benefits of research.^45,50^ To avoid the risk of under-representing this population, future research should identify ways to overcome these barriers. Since persons with dementia are often dependent on family caregivers, it would be meaningful to recruit both persons with dementia and family caregivers.

Clinical contact persons proved to play an important role in recruitment.^8^ Our review also suggests that clinical contact persons and the situation in clinical facilities influence or even hamper the recruitment of persons with dementia. For example, clinical contact persons occasionally deny the relevance of involving persons with dementia in studies. A negative attitude towards research is not uncommon.^4,42^ These kinds of barriers may be a problem for researchers, as they cannot control or influence the situation of clinical contact persons. Therefore, it is important that researchers consider the situation in nursing homes or clinics with regard to potential collaborations. For example, study information sessions in small groups before and after a study could be helpful for obtaining consent from clinical contact persons.^60^

The results of our review showed that comprehensible study information and its appropriate presentation are important aspects of recruitment. Inadequate or incomplete communication can hinder recruitment efforts. This seems particularly relevant since persons with dementia often have problems with speech and vision. Therefore, easy-to-understand language^61,62^ and appropriately prepared documents prove to be particularly important for building relationships and motivating participation.^9^ Studies and meta-research addressing target-group-specific language in study materials could offer helpful information.

The identified facilitators and barriers may provide a basis to develop effective strategies for recruiting persons with dementia. It should be noted that the identified recruitment strategies tend to take less account of the specific dementia experience. Our results highlight the need for future research aiming to develop and to test dementia-specific recruitment strategies.^9^

Study recruitment contains multiple steps: identifying and approaching potential participants, informing them about a study, and obtaining their informed consent.^63^ Identifying and approaching potential participants is a prerequisite for obtaining consent to take part in a study.^63,64^ The success or failure of study recruitment, however, may be part of a complex process depending on the attitude of society towards dementia. Public awareness plays an important role.^65^ Efforts to open up research and to allow public involvement in research are essential.^66^ Therefore, it is challenging to consider study recruitment as a complex intervention and take into account all related variables.

The studies included in this review assessed several strategies to identify study participants. Commonly measured outcomes were the number and rate of participants recruited. Further analysis is necessary to determine to what extent these outcomes indicate the success of recruitment strategies and what they reveal about identifying, approaching, and consenting to dementia care studies.

To develop recruitment-related research and to optimize the design of recruitment studies we consider it important that researchers answer the following questions:

Which recruitment strategies did they use? Why and how did they use these strategies?

Which outcome did they anticipate? What was the target group they intended to reach?

Which intervention did they use to meet their target-group? Who ultimately agreed to participate in the research project?

These questions may help to provide more precise information about recruitment processes.

They also allow to reflect researchers’ context and perspectives.

To answer our review questions, we exclusively identified cross-sectional and qualitative studies with unclear representativeness of the target population. In addition and even more alarming is the fact that there were no prospective interventional studies in which potential participants were assigned to one or more recruitment strategies that were comparatively assessed. Therefore, it is not possible to draw causal conclusions about the effectiveness of the identified recruitment strategies. This underlines the need for more robust study designs in this area of research.

To design future studies on the recruitment of persons with dementia, “Studies Within A Trial” (SWAT) may be helpful as a methodological concept. SWAT is a “a self-contained research study that has been embedded within a host trial with the aim of evaluating or exploring alternative ways of delivering or organising a particular trial process”.^67^ From our point of view, SWAT can be used in any study design. It is suitable to investigate recruitment processes in any host study design. It should not be limited to randomised trials. Consequently, SWAT can help to develop, test, and refine recruitment strategies for study designs of choice, e.g. clinical trials, observational studies, and qualitative studies. Thus, it can contribute to a shared and optimized understanding of recruitment in dementia care studies.^9^ However, clarifications are necessary in advance. Additional costs and ethical relevance should be considered.

Compared to other reviews, the number of included studies seems to be rather small.^21,22,68^ However, we assume that the identified study landscape is based on an increased risk of publication bias and only in a fraction of studies recruitment outcomes are assessed and reported.^69^ We also assume that authors published the corresponding results only when recruitment was successful. Therefore, we would like to encourage authors to assess recruitment processes and to report the related information in a transparent way. This is an important prerequisite to inform the design of future recruitment research.

### Limitations

Our review has several limitations. First, we conducted a specific search in major biomedical and care-related bibliographic databases. Therefore, we may have missed studies not indexed in these sources. However, our approach also included searches in a database dedicated to recruitment studies (ORRCA). We also applied citation searching. Thus, we assume a low risk of missed studies that would have influenced our results and conclusions. Second, one reviewer screened titles and abstracts for eligibility and extracted data –confirmed by a second reviewer, if necessary. This reviewer was very experienced in reviewing dementia-related and methodological studies. He ensured careful screening (inclusion of full-text screening in case of uncertainty or confirmation by a second reviewer). Consequently, we assume a low risk of wrongly excluded studies and data extraction errors. Third, to our knowledge, there is no standardized and common definition of dementia care studies. To ensure a unified selection of eligible studies, we discussed borderline cases within the review team. Fourth, we did not extract information on the development of recruitment strategies and on the involvement of persons with dementia in the design. This information may have underlined the need for dementia-specific development of recruitment strategies, their testing and refinement. This should be considered in a review update. Finally, we restricted the review to dementia care studies. This allowed us to consider a wide range of study designs and purposes. Future reviews may take into account a comparison of findings on recruitment in dementia care studies with research following a (bio)medical focus. On this basis, it will be possible to determine whether there are differences regarding facilitators, barriers, and strategies for recruitment.

## Conclusions

There is limited evidence on the effectiveness of recruitment strategies in dementia care studies. Our analysis of facilitators and barriers highlights the situation of participating persons with dementia, researchers, and clinical contact persons. It may inform and guide research teams in designing strategies to identify persons with dementia for recruitment to dementia care studies.

## Supplemental Material

Supplemental Material - Recruiting Persons With Dementia: A Systematic Review of Facilitators, Barriers, and Strategies Supplemental Material for Recruiting Persons With Dementia: A Systematic Review of Facilitators, Barriers, and Strategies by Julian Hirt, Thomas Beer, Stefano Cavalli, Stefano Cereghetti, Elia R.G. Pusterla and Adelheid Zeller in American Journal of Alzheimer's Disease & Other Dementias®

## Data Availability

All data generated and analyzed in this study is part of the publication and appendix.*
