# Supplementary material for: Recruiting Persons With Dementia: A Systematic Review of Facilitators, Barriers, and Strategies
Source: Am J Alzheimers Dis Other Demen. 2024 Aug 13;39:15333175241276443. doi: 10.1177/15333175241276443 (PMC11322929; doi:10.1177/15333175241276443)
Supplement: Supplemental Material - Recruiting Persons With Dementia: A Systematic Review of Facilitators, Barriers, and Strategies [file sj-pdf-1-aja-10.1177_15333175241276443.pdf]

Recruiting persons with dementia: A systematic review of facilitators, barriers, and strategies

Hirt et al.

SUPPLEMENTAL APPENDIX

Supplemental Appendix 1. Database-specific search strategies

Supplemental Appendix 2. Extracted data of individual studies

Supplemental Appendix 3. Critical appraisal of the included studies

Supplemental Appendix 4. Individual strategies, outcomes, and results

Supplemental Appendix 1. Database-specific search strategies

MEDLINE/PubMed

June 27, 2022; 862 hits

((patient selection[MeSH Terms] OR recruit*[Title] OR particip*[Title] OR attendanc*[Title] OR assign*[Title] OR enrol*[Title] OR non-respond*[Title] OR respond*[Title] OR partner[Title] OR engagement[Title] OR involve*[Title] OR particip*[Title])

AND (research*[Title] OR stud*[Title] OR trial*[Title] OR survey*[Title] OR interview*[Title] OR observation*[Title])) AND (Dementia[MeSH Terms] OR dement*[Title] OR alzheimer*[Title])

CINAHL

June 27, 2022; 478 hits

((MH patient selection OR TI recruit* OR TI particip* OR TI attendanc* OR TI assign* OR TI enrol* OR TI non-respond* OR TI respond* OR TI partner OR TI engagement OR TI involve* OR TI particip*)

AND (TI research* OR TI stud* OR TI trial* OR TI survey* OR TI interview* OR TI observation*)) AND (MH Dementia OR TI dement* OR TI alzheimer*)

Web of Science Core Collection

June 27, 2022; 673 hits

((TI=recruit* OR TI=particip* OR TI=attendanc* OR TI=assign* OR TI=enrol* OR TI=non-respond* OR TI=respond* OR TI=partner OR TI=engagement OR TI=involve* OR TI=particip*)

AND (TI=research* OR TI=stud* OR TI=trial* OR TI=survey* OR TI=interview* OR TI=observation*)) AND (TI=dement* OR TI=alzheimer*)

Supplemental Appendix 2. Extracted data of individual studies

a. Studies included to answer review question 1 on barriers and facilitators to identifying and approaching people with dementia for recruitment to dementia care studies (n=11)

Reference<br><br>Year<br><br>Country	Aim	Study design	Setting	Sample: severity of dementia	Sample: type/perspective	Sample: n individuals	Data collection methods	Data analysis methods	Results
Benson [40]<br><br>2021<br><br>US	To understand perspectives and preferences regarding research recruitment of people with dementia and caregivers.	Qualitative study	Hospital or home care	Not specified; capacity to consent to research	Caregivers and/or people with dementia	31	Semi-structured interviews	Thematic analysis (following Braun and Clarke)	Facilitators (identified as recommendations):<br><br>Role ambiguity:<br>- Remove any badges from research staff that are similar in branding or style to the care staff<br>- Teach research staff to engage language that makes the distinction between care and research more explicit<br>- Offer the opportunity to follow-up and consent to the research after discharge from the acute illness care setting to signal a separation from the clinical setting and care<br>- Informed consent document and script emphasizing that participation in and withdrawal from the research study has no impact on care received during acute illness care<br><br>Motivational misconceptions in research:<br>- First investigated participant's understanding of the concept of research prior to further questioning motivations driving participation<br>- Ensure to inform that the study offers no direct benefits for caregivers or individuals with dementia and make sure to re-explain the research study purpose and benefit-to-risk ratio at the time of participation<br><br>Decision-making processes:<br>- Emphasize including the individual with dementia in the conversation by speaking to them along with the caregiver, rather than speaking about them in their presence, even if they themselves can not be recruited into the study due to lack of decisional capacity<br>- When more than one caregiver is present in the room, frame information about the study and participation broadly, inviting both caregivers to participate in the study if appropriate (since legally authorized representatives are not always the closest relative or friend to the individual with dementia)<br>- Offer time to individuals with dementia to consult with other important decision-makers prior to making a decision about participating in the study<br><br>Under-referral:<br>- Principal investigator informing on the heterogeneous nature of abilities, strengths, and limitations that may be experienced by individuals with dementia to each clinical recruitment partner<br><br>Strategies to improve relationship-building:<br>- Work with nursing staff to determine the most appropriate time for the study team to approach the individual with dementia, to anticipate and minimize disruptions during initial conversations<br>- Confirm whether caregivers are present in the room with the individual with dementia, and if the individual with dementia has decisional capacity, or has a legally authorized representative (proxy) decision maker<br><br>Data Collection Material Revisions:<br>- Create a list of phrases for interviewers to use before and after questions to acknowledge the difficulty of certain questions and allow participants to skip or come back to other questions, including, "I know it can be challenging to think of examples sometimes, would you like to come back to this question?" and, "Sometimes this question can be challenging for people, let me know if you prefer to skip it."
Chambers [41]<br><br>2017<br><br>Canada	To develop a research recrutiment guide and assess its use by provincial and local Alzheimer Societies.	Mixed-methods study	Not specified	Not applicable	Representatives and researchers (scoping review and stakeholder consultation)<br><br>Alzheimer society members (expert survey)	44 documents (scoping review: 16 studies, 11 tools, 17 websites; stakeholder consultation: 11 stakeholder)<br><br>55 Alzheimer society members from 20 societies (expert survey)	Scoping review and semi-structured interviews<br><br>Expert survey	No information (scoping review and semi-structured interviews)<br><br>Descriptive statistics (expert survey)	Facilitators (identified as recommendations):<br><br>- Meet people where they are such as community service centres, physician's offices and online<br>- Utilize peer leaders or champions to embed the discussion about research participation into service delivery
Davies [42]<br><br>2014<br><br>UK	To develop a national network of research-ready care homes to increase research activity and capacity in England.	Cross-sectional study	Care home	Not applicable	Researchers	21	Email request	No information	Facilitators:<br>- Producing a brochure outlining the study and its potential benefits<br>- Involving relatives in the research process as much as possible<br>- Using a designated team to work with the care home residents and staff, drawing on clinicians that were linked to the care homes<br>- Building in some research training for care home staff into the study process<br><br>Barriers:<br>- Studies that require a large time commitment from care home staff<br>- Staff turnover – some care homes that agreed to take part in studies dropped out after a change of management<br>- Care home organisations not giving permission for studies to be conducted in their care homes<br>- Research protocols that do not fit with the care home's mode of operation
Friz [43]	To examine views, preferences, and	Qualitative study	Hospital	Not specified; capacity to	Caregivers and/or people with	31	Semi-structured individual and	Thematic analysis (following Braun	Facilitators (identified as recommendations):

2021<br><br>US	recommendations regarding acute care-based Alzheimer's disease and related forms of dementia and caregivers research recruitment among persons with dementia and their caregivers.			participate	dementia		dyadic interviews	and Clarke)	Research Staff Attributes and Skills:<br><br>- Positive personal attributes among research staff desired<br><br>- Adaptive communication skills critical for recruitment<br><br>- Detailed and clear communication needed central to recruitment approach and description of study<br><br>- Importance of having a conversation and building a relationship to successful recruitment<br><br>Situation and timing:<br><br>- Reason for and context of a person's acute care situation including related acute care work processes are important considerations<br><br>- Periods of uncertainty may pose challenges for recruitment and decisions to participate<br><br>- Recruiters must consider person with dementia's patterns in cognition and preferred timing<br><br>Role of people with dementia:<br><br>- Inclusion of persons with dementia in conversations about research participation viewed as important, though perspectives on how differed<br><br>General perspectives on research:<br><br>- Varying familiarity with and perspectives on research may influence acute care recruitment preferences<br><br>- Shared personal motivations for research participation may influence acute care research recruitment preferences
Goodman [44]<br><br>2011<br><br>UK	To describe factors that support and inhibit recruitment and participation of people with dementia living in care homes.	Mixed-methods study	Care home	Not specified; capacity to participate	Caregivers, people with dementia, and care home staff	Not specified (241 individuals with dementia eligible for participation and 133 recruited in total)	Document analysis (recruitment process data) and field notes	No information	Facilitators:<br>- Engagement with the study was informed by the willingness of care home managers to talk about the study topic<br>- Care home staff's beliefs about how much people with dementia should and could be included in research, and attitudes towards the possibilities of discussing dying in a care home environment prefigured and influenced decisions about researchers' access to the older people, and how the conversations and explanations about the study were organised with residents, staff and family members<br>- Study information via meetings on weekends with residents as a group and their relatives present<br>- Released care home staff to support the recruitment process
Lee [45]<br><br>2022<br><br>Canada	To understand clinician attitudes and the barriers that impede research recruitment from specialized primary care-based memory clinics.	Cross-sectional study	Memory clinic	Not applicable	Clinicians	206	Survey	Descriptive statistics	Barriers:<br><br>Limited time/workload and resources for recruitment:<br><br>- Limited time within clinic assessment appointment to introduce studies<br><br>- Additional workload a burden on already stressed resources<br><br>- Resources needs - space, trained staff, time<br><br>Patients and caregivers belong too overwhelmed by the diagnosis/clinic appointment to process research information:

									- Clinic appointment being already overwhelming, so adding research recruitment adds to the emotional burden<br><br>- Time of diagnosis is distressing; they may be unable to process information about research and it would not be a priority, particularly if patient denies the diagnosis<br><br>Time, distance, transportation and cost for research participation:<br><br>- Long distances to research centers, particularly in rural and remote areas<br><br>- Transportation needs<br><br>- Costs for travel and transportation<br><br>Limited Information about research studies to share with patients and caregivers:<br><br>- Lack of information about studies and expectations of patients, such as time commitment<br><br>- Lack of understandable information about studies<br><br>- Lack of Information about potential benefits of participation<br><br>Perception of potential conflict of interest or coercion<br><br>- Potential for perceived conflict of interest between research recruitment and provision of care<br><br>- Unspecified 'conflict of interest'<br><br>Patient competency to consent:<br><br>- Concern that patients may not have the capacity to provide informed consent<br><br>Lack of knowledge about/experience with research among team members:<br><br>- Memory clinic team members lack of understanding of what research is, available studies, how to recruit for research, and how to explain research studies to potential participants
McPhillips [46]<br><br>2022<br><br>US	To explore factors that influence dyads' decision to enroll in a clinical trial.	Qualitative study; part of a RCT	Community	Not specified; capacity to participate in parent study (RCT)	Dyads (people with dementia and caregivers)	21	Semi-structured interviews	Content analysis	Facilitators:<br><br>Attitudes toward joining:<br>- Personal desires of wanting to learn from being in the study<br>- Positive experiences of in-person meetings with knowledgeable staff during recruitment events<br>- Financial incentive with joining the study<br><br>Subjective norm:<br>- Happiness to help their study partner who needed a second person to participate (dyad recruitment)
Prick [47]<br><br>2014<br><br>The Netherlands	To study the quality of the success rate of recruitment.	Cross-sectional study; part of a RCT	Community	Not specified; mini-mental state examination score <14 and receiving more than 2 days of respite care in a day-care facility	Dyads (people with dementia and caregivers)	22	Semi-structured interviews	Thematic analysis (not specified)<br><br>Descriptive statistics	Barriers:<br>- Intensity of the intervention study<br>- Expected participation burden for the caregiver<br>- Lack of time of the caregiver<br><br>Facilitators:<br>- Possibility to exercise during the intervention<br>- Advantages of a 'home-based' program

									- Participation in scientific research and to do something for society
Robinson [48]<br><br>2020<br><br>US	To describe the development of a set of storytelling materials to be used in a sequential cohort investigation of the impact of a culturally informed narrative campaign on the recruitment into Alzheimer's research.	Qualitative study	Community	Not specified; Montreal Cognitive Assessment scores of 18 or lower	Caregivers and/or people with dementia	25	Focus groups	Thematic analysis (following Krueger)	Facilitators (identified as recommendations):<br><br>- Avoid “us versus them” perceptions of the researcher/participant relationship by recognizing people’s existing knowledge about dementia<br><br>- Acknowledge potential participants’ personal experience with or concerns about dementia<br><br>- Tell stories that emphasize both the “big picture” collective benefits and the personal benefits of participation in the research<br><br>- Leverage the positive experiences past research participants have had with the research staff<br><br>- Avoid medical and scientific jargon when talking with research recruits and participants<br><br>- Use strong explanatory chains to build understanding of the research process<br><br>- Make data meaningful to non-experts through social math<br><br>- Choose visuals and stories that represent equity in all aspects of the research<br><br>- Find appropriate ways to use past research participants as messengers
Thompson [49]<br><br>2021<br><br>Australia	To explore ways that music therapy researchers have previously included people with dementia in qualitative interviews, and how practices can be improved to be more inclusive and accessible.	Qualitative study	Community	Not applicable	Researchers and caregivers	7	Semi-structured interviews	Thematic analysis (following Braun and Clarke)	Facilitators (identified as recommendations):<br>- Offer of ‘multiple interviews’ option – participants can select to do multiple interviews over time (including interviews in different formats if they wish, such as a focus group interview, followed by an individual interview)<br>- Gathering information about participant preferences (including communication styles, support/access needs) through discussion with participants and/or music therapists facilitating the choirs (prior to interviews)<br>- Offering options for mode of interview (e.g. focus group, individual or dyad)<br>- Preparing information to provide to caregivers as to how they could be supportive during the interview (if required)
Williams [50]<br><br>2001<br><br>US	To explore strategies to overcome the barriers for obtaining family consent for participation in Alzheimer’s research.	Cross-sectional study	Nursing home	Not specified	Caregivers	Not specified	Document analysis (recruitment process data)	No information	Barriers:<br>- Resistance to any attempt to interfere with their loved one’s rest expressed by family members<br>- Belief was that it would be best for the elder to be left alone<br>- Belief that nothing could or should be done to improve the quality of life for this individual with dementia<br><br>Facilitators:<br>- Discussions of attitudes and beliefs among the members of the research team prior to approaching families for consent<br>- Avoid the implication that the research protocol would mean care superior to that which was provided at the nursing home<br>- Expect a lengthier process and offer to speak to other family members by making an appointment either to meet at the nursing home or to make a home visit<br>- Recruitment calls to caregivers in the evening or on Sunday afternoon<br>- Meet with caregivers in person

Abbreviations: UK = United Kingdom; US = United States.

b. Studies included to answer review question 2 on the effectiveness of recruitment strategies in dementia care studies (n=10)

**Reference**<br><br>**Year**<br><br>**Country**	**Aim**	**Study design**	**Setting**	**Sample: severity of dementia**	**Sample: n individuals**	**Recruitment strategy**	**Comparison**	**Outcome and measurement**	**Results**
Beattie [51]<br><br>2018<br><br>Australia	To analyze and compare recruitment strategies used within nine separate studies conducted by the same research team.	Various	Various	Various	Various	Community/Health Care Outreach<br><br>- Partnership with service provider<br><br>- Service providers assisted with recruitment<br><br>- Direct contact with potential participants<br><br>Social Marketing<br><br>- Flyers and posters<br><br>- Mass media<br><br>- Online marketing<br><br>Other Strategies<br><br>- Minimize participant burden<br><br>- Provision of incentive to participate<br><br>- Referral from other studies and past participants	Multiple strategies	Monetary cost<br><br>Recruited participants relative to the total number of participants desired for the project (Effectiveness)	Cost/Effectiveness among nine studies:<br><br>Community/Health Care Outreach<br><br>- Partnership with service provider: moderate to high cost / somewhat to highly effective<br><br>- Service providers assisted with recruitment: low to high cost / somewhat to highly effective<br><br>- Direct contact with potential participants: low to high cost / no participants to moderately effective<br><br>Social Marketing<br><br>- Flyers and posters: low to high cost / no participants to moderately effective<br><br>- Mass media: low to high cost / no participants to highly effective<br><br>- Online marketing: low to moderate cost / no participants to moderately effective<br><br>Other Strategies<br><br>- Minimize participant burden: low cost / somewhat to moderately effective<br><br>- Provision of incentive to participate: no to moderate cost / somewhat effective<br><br>- Referral from other studies and past participants: low cost / no participants to somewhat effective
Birkenhäger-Gillesse [52]<br><br>2021<br><br>The Netherlands	Process evaluation to estimate recruitment activities.	Cross-sectional study; part of a RCT	Holiday accommodation	Not specified	Referral: 200<br>Participation: 142	- Newsletter of the Dutch Alzheimer Association<br>- Advertisement in local newspaper<br>- Facebook post on Dutch Alzheimer Association<br>- News article in local newspaper	Multiple strategies	Participation rate	Participation rate increased considerably but temporary after<br>- Newsletter of the Dutch Alzheimer Association<br>- Facebook post on Dutch Alzheimer Association<br>- Advertisement article in local newspaper<br><br>No increase after news article in local newspaper
Davies [42]<br><br>2014<br><br>UK	To evaluate a national network of research-ready care homes to increase research activity and capacity in England in its first year.	Cross-sectional study	Care home	Not applicable	141	- Building on prior working relationships with care homes<br>- Cold calling with follow up, group meetings for and visits of care homes	Not applicable	Joining a care home research network	- Only 1/4 sites achieved the target recruitment of 10 care homes and did this in three months; this site had a pre-existing working relationship with care homes and had maintained contact them through phone calls, newsletters and invitations to training and events<br>- Cold calling to care homes that had no existing relationships with the sites, with follow up, group discussions and visits were labour and resource intensive for example, staff in one pilot site made 55 phone calls in order to recruit four care homes
Field [53]<br><br>2019<br><br>UK	To describe the challenges of recruiting people with dementia, using experiences from one recently completed trial as	Cross-sectional study; part of a RCT	Community	Mild to moderate	Referral: 90 (site A); 80 (site B); 60 (site C)<br><br>Participation: 83 (site A); 73 (site B);	Site A/B/C (within-/non-National Health System sites):<br><br>- Direct referral by memory services clinicians<br><br>- Attendance at psychosocial intervention groups by researchers	Multiple strategies	Referral to participation rate<br><br>Length of time taken to recruit	Referral to participation rate:<br><br>- Site A: 25%<br><br>- Site B: 31%

	an exemplar.				73 (site C)	- Leaflets and posters displayed<br><br>- Occupational therapists delivering the intervention identifying potential participants<br><br>- Join Dementia Research (JDR): an online resource that enables people to register interest in participating in dementia research and thereby be 'matched' to relevant studies.<br><br>Additional strategies (site A):<br><br>- Attendance at clinical team business meetings by researchers<br><br>- Research team made contact with people who had participated in other studies previously and had agreed to be contacted about future studies<br><br>- Attendance at community groups by research staff<br><br>- One mail out via non--statutory sector organisation / sending non--statutory sector organisation staff study information<br><br>Additional strategies (site B):<br><br>- Pre-screening of clinical records by a research nurse<br><br>- Ad--hoc mail outs targeting potentially eligible participants choosing to attend follow--up appointments offered at local GP practices, instead of memory services at the hospital<br><br>- Leaflets and posters displayed (other NHS Trust locations)<br><br>- Information displayed in GP practices associated with memory services<br><br>- Patient Identification Centre in another NHS Trust<br><br>Additional strategies (site C):<br><br>- Potential participants identified by within multidisciplinary clinical meetings<br><br>- Attendance at clinical team business meetings by researchers<br><br>- Leaflets and posters displayed (other NHS Trust locations)<br><br>- Research team made contact with people who had participated in other studies previously and had agreed to be contacted about future studies<br><br>- Attendance at community groups by research staff<br><br>- One mail out via non--statutory sector organisation / sending non--statutory sector organisation staff study information			- Site C: 51%<br><br>Length of time taken to recruit:<br><br>- Site A: 29 months<br><br>- Site B: 29 months<br><br>- Site C: 11 months
Greimel [54]<br><br>2022<br><br>US	To describe recruitment and screening processes and results.	Cross-sectional study; part of a RCT	Community	Mild to moderate	96	- Clinical and word-of-mouth referrals<br><br>- Alzheimer's Association (AA) services and events<br>- Flyers/brochures<br>- Presentations at the university and medical centers, senior centers, and community events<br>- Targeted mailings<br>- Advertisements<br>- Study website, social media, and research registries<br>- Non-AA community groups<br>- Press releases/newsletters/newspaper article	Multiple strategies	Number of randomized participants per strategy<br><br>Monetary cost	Randomized participants per strategy; monetary cost per randomized participant:<br>- Clinical and word-of-mouth referrals Alzheimer's: 28%; $0<br>- Association (AA) services and events: 22%; $734<br>- Flyers/brochures: 13%; $625<br>- Presentations: 12%; $368<br>- Targeted mailings: 8%; $290<br>- Advertisements: 3%; $1719<br>- Study website, social media, and research registries: 3%; $225<br>- Non-AA community groups: 2%; $793<br>- Press releases/newsletters/newspaper article: 1%; $270

									Total monetary cost per randomized participant: $398
Heward [55]<br><br>2022<br><br>UK	To examine the effectiveness of recruitment and retention strategies in people with dementia.	Cross-sectional study; part of a RCT	Community	Mild to moderate	Referral: 359<br>Participation: 86	- National Health Service Trusts and/or Join Dementia Research database<br>- General Practitioner Participant Identification<br>- Memory Support and Advisory Service database<br>- Public relations campaign	Multiple strategies	Number of randomized participants per strategy<br><br>Referral to randomization rate	Randomized participants per strategy; referral to randomization rate:<br>- National Health Service (NHS) Trusts and/or Join Dementia Research database: 62%; 20%<br>- General Practitioner Participant Identification: 14%; 32%<br>- Memory Support and Advisory Service database: 2%; 67%<br>- Public relations campaign: 20%; 32%<br>- Unknown: 1%; 33%<br><br>Total referral to randomization rate: 24%
Morrison [56]<br><br>2016<br><br>US	To evaluate the yield and cost of recruitment strategies.	Cross-sectional study; part of a RCT	Community	Not specified; Mini-Mental State Examination >0	Referral: 284<br><br>Participation: 237	- Direct mailing<br><br>- Advertisement in local newspaper<br><br>- Community outreach (talks to regional aging organizations, informational booths at health fairs, and consumer-oriented conferences, and distributing study brochures to local public libraries and employees of the academic institution)	Multiple strategies	Number of participants per strategy<br><br>Referral to participation rate<br><br>Monetary cost per participant	Participants per strategy; referral to participation rate; monetary cost per participant:<br><br>- Direct mailing: 57%; 85%; $63<br><br>- Advertisement in local newspaper: 26%; 85%; $224<br><br>- Community outreach: 17%; 75%; $350<br><br>Total referral to participation rate; monetary cost per participant: 83%; $154
Prick [47]<br><br>2014<br><br>The Netherlands	To study the quality of the success rate of recruitment.	Cross-sectional study; part of a RCT	Community	Not specified; mini-mental state examination score <14 and receiving more than 2 days of respite care in a day-care facility	No information	- Advertisements in national and local newspapers and on geriatric websites<br>- Personal letters sent to caregivers of people with dementia via caregiver organizations<br>- Personal approach to the dyads by giving presentations at local Alzheimer cafes (public meetings for people with dementia, their caregivers and others)	No formal comparison reported	Recruitment rate	Recruitment rate:<br>- Advertisements in national and local newspapers and on geriatric websites: almost no reaction from interested dyads<br>- Personal letters sent to caregivers of people with dementia via caregiver organizations: almost no reaction from interested dyads<br>- Personal approach to the dyads by giving presentations at local Alzheimer cafes (public meetings for people with dementia, their caregivers and others): was more successful
Robinson [48]<br><br>2020<br><br>US	To describe the development of a set of storytelling materials to be used in a sequential cohort investigation of the impact of a culturally informed narrative campaign on the recruitment into Alzheimer's research.	Qualitative study	Community	Not specified; Montreal Cognitive Assessment scores of 18 or lower	25	- Community outreach<br>- Doctor's office<br>- Family<br>- Other research studies<br>- Other	Multiple strategies	Referral source	Referral source:<br>- Doctor's office: 7 (28%)<br>- Community outreach: 6 (24%)<br>- Other research studies: 3 (12%)<br>- Family: 2 (8%)<br>- Other: 2 (8%)<br>- Unknown: 5 (20%)
Samus [57]<br><br>2015<br><br>US	To provide a critical review of a recruitment approach used to identify, recruit and enroll a diverse community-based sample of persons with memory disorder.	Cross-sectional study; part of a RCT	Community	Not specified	Referral: 1275<br>Participation: 303	- Community liaison organizations<br>- Community organizations letters<br>- Community organizations displaying/providing flyers/brochures/bookmarks<br>- University research registry<br>- General community outreach	Multiple strategies	Number of participants per strategy<br><br>Referral to participation rate	Number of participants per strategy; referral to participation rate<br>- Community liaison organizations: 29%; 49%<br>- Community organizations letters: 40%; 17%<br>- Community organizations displaying/providing flyers/brochures/bookmarks: 6%; 39%<br>- University research registry: 5%; 9%<br>- General community outreach: 21%; 35%<br><br>Total referral to participation rate: 24%

Abbreviations: UK = United Kingdom; US = United States.

Supplemental Appendix 3. Critical appraisal of the included studies (clustered by review question and study design)

**REVIEW QUESTION #1 (n=11)**										
**Cross-sectional studies (n=5)**	**#1: Is the sampling strategy relevant to address the research question?**	**Quote or comment to justify #1**	**#2: Is the sample representative of the target population?**	**Quote or comment to justify #2**	**#3: Are the measurements appropriate?**	**Quote or comment to justify #3**	**#4: Is the risk of nonresponse bias low?**	**Quote or comment to justify #4**	**#5: Is the statistical analysis appropriate to answer the research question?**	**Quote or comment to justify #5**
Davies [42]	Yes	The source of sample was relevant to or taken from the target population.	Can't tell	No information on non-responders.	Can't tell	Data collection instrument and related details were not provided.	Can't tell	No information on non-responders.	Yes	Descriptive data provided.
Lee [45]	Yes	The source of sample was relevant to the target population.	Can't tell	No information on non-responders.	Can't tell	Data collection instrument and related details were not provided.	Can't tell	No information on non-responders.	Yes	Descriptive data provided.
Prick [47]	Yes	The source of sample was relevant to or taken from the target population.	Yes	Barriers and facilitators were derived from participants and non-participants of the parental study (randomized trial).	Yes	"We structured and evaluated our process evaluation of the RCT study and the intervention according to the framework presented by Reelick and colleagues."	Can't tell	No information on sociodemographics of non-responders.	Yes	Descriptive data provided.
Williams [50]	Yes	The source of sample was relevant to or taken from the target population.	Yes	Barriers and facilitators were derived from participants and non-participants of the parental study (randomized trial).	Can't tell	Data collection instrument and related details were not provided.	Can't tell	No information on sociodemographics of non-responders.	Yes	Descriptive data provided.
**Qualitative studies (n=5)**	**#1: Is the qualitative approach appropriate to answer the research question?**	**Quote or comment to justify #1**	**#2: Are the qualitative data collection methods adequate to address the research question?**	**Quote or comment to justify #2**	**#3: Are the findings adequately derived from the data?**	**Quote or comment to justify #3**	**#4: Is the interpretation of results sufficiently substantiated by data?**	**Quote or comment to justify #4**	**#5: Is there coherence between qualitative data sources, collection, analysis and interpretation?**	**Quote or comment to justify #5**
Benson [40]	Yes	"… investigating ADRD patient and caregiver views on research recruitment and participation."	Yes	"Interviews followed a semi-structured interview guide ..."	Yes	-	Yes	-	Yes	-
Friz [43]	Yes	"… to elicit and examine the views, preferences, and recommendations of patients with ADRD and their caregivers regarding acute carebased recruitment for ADRD research …".	Yes	"Interviews were designed to: (1) elicit participants' perspectives in response to invitations to participate in an ADRD study (recruitment practices) in the acute care context, (2) identify factors participants weighed when deciding to participate, (3) probe perceived readiness, barriers, and facilitators, and (4) garner input on steps researchers should take when recruiting people with ADRD and their caregivers…"	Yes	-	Yes	-	Yes	-
McPhillips [46]	Yes	"…to explore factors that influence dyads' decision to enroll in a clinical trial of a nonpharmacological behavioral intervention to identify strategies for improving enrollment of dyads into clinical trials."	Yes	"During the interview, questions were asked from a semi-structured interview guide that was developed by the team based on the TPB constructs and the aim of the study."	Yes	-	Yes	-	Yes	-
Robinson [48]	Yes	"We conducted and analyzed semi-structured interviews to capture the research participation stories of African American/Black participants and study partners from the University of Pittsburgh's Alzheimer's Disease Research Center."	Yes	"Semi-structured interviews were conducted separately with participants and their study partners. Each lead question began broadly, for example, "Tell me about how you learned about the Alzheimer's Research Center," "Thinking back, what led you to consider participating in Alzheimer's research?" and "What has it been like to participate in	Yes	-	Yes	-	Yes	-

				Alzheimer's research?" …".						
Thompson [49]	Yes	"This study aimed to explore ways that music therapy researchers have previously included people with dementia in qualitative interviews, and how practices can be improved to be more inclusive and accessible."	Yes	"We used a semi-structured interview format, allowing for prompt questions about the participant's experiences, as well as broader topics should they come up during the course ofthe interview […]. A schedule of questions can be found in Supplemental online Supplemental Appendix."	Yes	-	Yes	-	Yes	-
**Mixed-methods studies (n=2)**	**#1: Is there an adequate rationale for using a mixed methods design to address the research question?**	**Quote or comment to justify #1**	**#2: Are the different components of the study effectively integrated to answer the research question?**	**Quote or comment to justify #2**	**#3: Are the outputs of the integration of qualitative and quantitative components adequately interpreted?**	**Quote or comment to justify #3**	**#4: Are divergences and inconsistencies between quantitative and qualitative results adequately addressed?**	**Quote or comment to justify #4**	**#5: Do the different components of the study adhere to the quality criteria of each tradition of the methods involved?**	**Quote or comment to justify #5**
Chambers [41]	Yes	"A mixed-method, iterative approach was undertaken to develop the Guide, organized by a research and development phase, and followed by a piloting phase."	Yes	"The first step of the research and development phase involved a scoping review of the literature and stakeholder consultations […]. The second step involved drafting the Guide as informed by findings from the review and stakeholder interviews, and collaborative review process to generate relevant organizational scenarios illustrating the support of research recruitment in practice. The piloting phase involved two rounds of review with a sample of Society offices across Canada. The goals of the piloting phase were to a) assess the content, design, and usability of the Guide; b) refine the Guide based upon user feedback; and c) determine the opinion of Society staff regarding potential for uptake and gather any early outcomes."	Yes	-	Can't tell	Not reported if there were divergences and inconsistencies between quantitative and qualitative results.	Yes	-
Goodman [44]	Yes	"It had a mixed-method design that employed both quantitative and qualitative methods of data collection."	Yes	"Once older people were recruited to the study, this included review of care notes at four monthly intervals, interviews with people with dementia, care home staff and health care professionals and documentary review of guidance and protocols on end-of-life care used by the care homes."	Yes	-	Can't tell	Not reported if there were divergences and inconsistencies between quantitative and qualitative results.	Yes	-
**REVIEW QUESTION #2 (n=10) ***										
**Cross-sectional studies (n=8)**	**#1: Is the sampling strategy relevant to address the research question?**	**Quote or comment to justify #1**	**#2: Is the sample representative of the target population?**	**Quote or comment to justify #2**	**#3: Are the measurements appropriate?**	**Quote or comment to justify #3**	**#4: Is the risk of nonresponse bias low?**	**Quote or comment to justify #4**	**#5: Is the statistical analysis appropriate to answer the research question?**	
Birkenhäger-Gillesse [52]	Yes	The source of sample was relevant to or taken from the target population.	Can't tell	No information on non-responders.	Yes	Participation rate by referral source.	Can't tell	No information on non-responders.	Yes	Descriptive data provided.
Davies [42]	Yes	The source of sample was relevant to or taken from the target population.	Can't tell	No information on non-responders.	Yes	Recruitment data.	Can't tell	No information on non-responders.	Yes	Descriptive data provided.
Field [53]	Yes	The source of sample was relevant to or taken from the target population.	Can't tell	No information on non-responders.	Yes	Referral to participation rate: "A secondary analysis of recruitment data routinely collected by …".	Can't tell	No information on non-responders.	Yes	Descriptive data provided.

Greimel [54]	Yes	The source of sample was relevant to or taken from the target population.	Can't tell	No information on non-responders.	Yes	Recruitment data and tracking recruitment costs (see chapter "Tracking Recruitment Costs").	Can't tell	No information on non-responders.	Yes	Descriptive data provided.
Heward [55]	Yes	The source of sample was relevant to or taken from the target population.	Can't tell	No information on non-responders.	Yes	Number of randomized participants per strategy and referral to randomization rate.	Can't tell	No information on non-responders.	Yes	Descriptive data provided.
Morrison [56]	Yes	The source of sample was relevant to or taken from the target population.	Can't tell	No information on non-responders.	Yes	Number of participants per strategy, referral to participation rate, and monetary cost per participant.	Can't tell	No information on non-responders.	Yes	Descriptive data provided.
Prick [47]	Yes	The source of sample was relevant to or taken from the target population.	Can't tell	No information on non-responders.	Yes	Recruitment rate.	Can't tell	No information on non-responders.	Yes	Descriptive data provided.
Samus [57]	Yes	The source of sample was relevant to or taken from the target population.	Can't tell	No information on non-responders.	Yes	Number of participants per strategy, referral to participation rate.	Can't tell	No information on non-responders.	Yes	Descriptive data provided.
**Qualitative studies (n=1)**	**#1: Is the qualitative approach appropriate to answer the research question?**	**Quote or comment to justify #1**	**#2: Are the qualitative data collection methods adequate to address the research question?**	**Quote or comment to justify #2**	**#3: Are the findings adequately derived from the data?**	**Quote or comment to justify #3**	**#4: Is the interpretation of results sufficiently substantiated by data?**	**Quote or comment to justify #4**	**#5: Is there coherence between qualitative data sources, collection, analysis and interpretation?**	
Robinson [48]	Can't tell	Findings from the study that were used in this systematic review are taken from interview sample characteristics.	Yes	-	Can't tell	Findings from the study that were used in this systematic review are taken from interview sample characteristics.	Can't tell	Findings from the study that were used in this systematic review are taken from interview sample characteristics.	Can't tell	Findings from the study that were used in this systematic review are taken from interview sample characteristics.

Abbreviations: ADRD = Alzheimer's disease and related dementias.

* One report on the evaluation of multiple studies was not critically appraised due to the lack of individual study details [51].

Supplemental Appendix 4. Individual strategies, outcomes, and results

Domain	Strategy	Outcome
**Study-related**	Incentives for participants	Recruited participants
		Overall cost
	Minimize participant burden	Recruited participants
		Overall cost
**Information, media, and advertisement**	***In person***	
	Presentations at the university and medical centers, senior centers, and community events	Recruited participants
		Overall cost
	Presentations at local Alzheimer cafes	Recruited participants
	Clinical and word-of-mouth referral	Recruited participants
		Cost per recruited participant
	Electronic	
	Online marketing	Recruited participants
		Overall cost
	Facebook post on Dutch Alzheimer Association	Recruited participants
	Targeted mailings	Recruited participants
		Cost per recruited participant
	Direct mailing	Recruited participants
		Referral to participation rate
		Cost per recruited participant
	Newsletter of the Dutch Alzheimer Association	Recruited participants
	Study website, social media, and research registries	Recruited participants
		Cost per recruited participant
	University research registry	Recruited participants
		Referral to participation rate
	National Health Service Trusts and/or Join Dementia Research database	Recruited participants
		Referral to randomization rate
	Memory Support and Advisory Service database	Recruited participants
		Referral to randomization rate
	Print	
	Flyers and posters	Recruited participants
		Overall cost
	Flyers and brochures	Recruited participants
		Cost per recruited participant
	Community organizations displaying/providing flyers, brochures, and bookmarks	Recruited participants
		Referral to participation rate
	Personal letters sent to caregivers of people with dementia via caregiver organizations	Recruited participants
	Advertisement in local newspaper	Recruited participants
	Advertisement in local newspaper	Recruited participants
		Referral to participation rate
		Cost per recruited participant
	News article in local newspaper	Recruited participants
	Various or not specified	
	Mass media	Recruited participants
		Overall cost
	Advertisements	Recruited participants
		Cost per recruited participant
	Advertisements in national and local newspapers and on geriatric website	Recruited participants
	Press releases, newsletters, and newspaper article	Recruited participants
		Cost per recruited participant
	Public relations campaign	Recruited participants
		Referral to randomization rate
	Alzheimer's Association services and events	Recruited participants
		Cost per recruited participant
	Non-Alzheimer's Association community groups	Recruited participants
		Cost per recruited participant
	Community outreach	Recruited participants
		Referral to participation rate
		Cost per recruited participant
	Community outreach	Referral source
	General community outreach	Recruited participants
		Referral to participation rate
	Community organizations letters	Recruited participants
		Referral to participation rate
	Community liaison organizations	Recruited participants
		Referral to participation rate
	Direct contact with potential participants	Recruited participants
		Overall cost
	Family	Referral source
	General practitioner	Recruited participants
		Referral to randomization rate
	Doctor's office	Referral source
**Networking and collaboration**	***With other study efforts***	
	Referral from other studies and past participants	Recruited participants

		Overall cost
	Other research studies	Referral source
	With clinical care provider	
	Partnership with service provider	Recruited participants
		Overall cost
	Service providers assisted with recruitment	Recruited participants
		Overall cost
	Building on prior working relationships with care homes	Joining a care home research [illegible]
**Multimodal strategies ***	Network and collaboration with clinical care providers and past and potential participants; in person, print, and electronic media and advertisements	Referral to participation rate
		Length of time taken to recruit
	Cold calling with follow up, group meetings for and visits of care homes	Joining a care home research [illegible]

Abbreviations: # = Reference number.

Notes: Beattie et al. [51] summarized the results of several studies; * Multimodal strategies cover several individual recruitment strategies of which results have not been reported separately.
